# AGING IN AMERICA: CARE AND COMMUNICATION ACROSS THE FIELD

**DOI:** 10.1093/geroni/igae098.3785

**Published:** 2024-12-31

**Authors:** Brianna Margulis, Alexandra Garcia, Richelle Matarazzo, Lynn Tepper, Carol Kunzel, Stacey Whalen

**Affiliations:** Columbia University Medical Center, New York, New York, United States; Columbia University, New York, New York, United States; Columbia School of Social Work, New York, New York, United States; Columbia University College of Dental Medicine, New York, New York, United States; Columbia University Medical Center, New York, New York, United States; Columbia University College of Dental Medicine, New York, New York, United States

## Abstract

Northern Manhattan has the largest population of individuals over 65. Students within the graduate programs of Columbia University electively participated in the workshop “Aging in America: Care and Communication Across the Field.” The workshop aimed to provide future professionals with communication techniques and best practices when caring for the aging population. Before and after participating in the workshop, participants completed a survey asking them to self-assess their readiness to communicate with the aging population. The workshop also taught participants how to recognize at-risk-for lower health literacy individuals and diminish barriers whenever possible. The workshop randomized participants into small groups composed of different participating programs (College of Dental Medicine, Programs in Physical Therapy, Programs in Occupational Therapy, School of Nursing, MD Program, Clinical Pastoral Education Program, Program in Genetic Counseling, Institute of Human Nutrition, Mailman School of Public Health, and School of Social Work). Participants facilitated discussions about how they would utilize the tools provided to navigate scenarios concerning older adults. The pre and post-surveys indicated that our efforts impacted participants by making them feel more prepared to communicate with an older adult who has experienced sensory, cognitive, physical, and/or behavioral changes. Participants reported that they learned more about their future colleagues’ scope of practice, which promotes better communication. The limitations of our workshop were within the smaller sample size of responses we received. Graduate programs should implement this workshop among health professional students in the future.

